# Transcriptomic Analysis of Glioma Based on IDH Status Identifies ACAA2 as a Prognostic Factor in Lower Grade Glioma

**DOI:** 10.1155/2020/1086792

**Published:** 2020-03-21

**Authors:** Chenxing Wu, Hongwang Song, Xiaojun Fu, Shouwei Li, Tao Jiang

**Affiliations:** ^1^Beijing Neurosurgical Institute, Capital Medical University, Beijing, China; ^2^Sanbo Brain Hospital, Capital Medical University, Beijing, China; ^3^Department of Neurosurgery, Beijing Tiantan Hospital, Capital Medical University, Beijing, China; ^4^Department of Emergency Medicine, Shengjing Hospital of China Medical University, Shenyang, China; ^5^Chinese Glioma Genome Atlas Network (CGGA), Beijing, China

## Abstract

**Background:**

Glioma is the most common and lethal tumor in the central nervous system (CNS). More than 70% of WHO grade II/III gliomas were found to harbor isocitrate dehydrogenase (IDH) mutations which generated targetable metabolic vulnerabilities. Focusing on the metabolic vulnerabilities, some targeted therapies, such as NAMPT, have shown significant effects in preclinical and clinical trials.

**Methods:**

We explored the TCGA as well as CGGA database and analyzed the RNA-seq data of lower grade gliomas (LGG) with the method of weighted correlation network analysis (WGCNA). Differential expressed genes were screened, and coexpression relationships were grouped together by performing average linkage hierarchical clustering on the topological overlap. Clinical data were used to conduct Kaplan–Meier analysis.

**Results:**

In this study, we identified ACAA2 as a prognostic factor in IDH mutation lower grade glioma with the method of weighted correlation network analysis (WGCNA). The difference of ACAA2 gene expressions between the IDH wild-type (IDH-WT) group and the IDH mutant (IDH-MUT) group suggested that there may be different potential targeted therapies based on the fatty acid metabolic vulnerabilities, which promoted the personalized treatment for LGG patients.

## 1. Introduction

Glioma is the most common tumor in the central nervous system (CNS) and is divided into four grades by the World Health Organization (WHO). More than 70% of WHO grade II/III gliomas were found to harbor IDH mutations which mainly included IDH1 mutations and IDH2 mutations [1]. IDH1 mutations occur at much higher incidences than IDH2 mutations in lower grade glioma (WHO grade II and III gliomas, LGG) [2]. And it is found that IDH1 mutations and IDH2 mutations are mutually exclusive in gliomas. The majority of IDH mutations involve the catalytic pocket of the enzyme. IDH mutations are heterozygous missense mutations. IDH mutations predominantly occur at arginine 132 resulting in substitutions, including R132H (most common, 88%), R132C, R132L, R132S, and R132G [1].

Normal IDH1/2 catalyzes the NADP^+^-dependent oxidative decarboxylation of isocitrate to *α*-ketoglutarate (*α*-KG), producing CO_2_ and NADPH in the process [3]. The common function of IDH1/2 active site mutations is a neomorphic enzyme activity that catalyzes the conversion of *α*-KG to R-2-hydroxyglutarate (R-2HG), while oxidizing NADPH [4]. It has become clear that IDH mutations are associated with many epigenetic and metabolic changes in gliomas [5]. IDH mutations are thought to play a significant role in early tumorigenesis and precede other oncogenic mutations [6]. Although it is not clear which differences can be directly attributed to the IDH status, IDH wild-type and IDH mutant gliomas are two subtypes with different biological characteristics. And the IDH status was introduced into the 2016 CNS World Health Organization (WHO) classification [7]. Patients with IDH mutant gliomas had a better outcome than those with IDH wild-type genes [1]. However, as far as we know, patients with IDH mutant glioma exhibited the heterogeneous clinical outcomes. Over the past decade, genetic and molecular studies have identified several diagnostic and prognostic markers to stratify patients with IDH mutant glioma. Clinical data have also shown that some IDH mutant gliomas also have a poor prognosis as those with IDH wild-type gliomas, suggesting that there may be some different biological characteristics among the IDH mutant gliomas.

In this study, we identified ACAA2 as a prognostic factor in IDH mutation lower grade glioma with the method of weighted correlation network analysis (WGCNA). The difference of ACAA2 gene expressions between the IDH-WT group and the IDH-MUT group suggested that there may be different potential targeted therapies based on the fatty acid metabolic vulnerabilities, which promoted the personalized treatment for LGG patients.

## 2. Methods and Materials

### 2.1. Patients

The gene expression RNA-seq normalized data of LGG samples with IDH status and clinical information from The Cancer Genome Atlas (TCGA) were downloaded from the cBioPortal (http://www.cbioportal.org) [8] and the UCSC Xena (https://tcga.xenahubs.net). Messenger RNA microarray and RNA-seq normalized data from the Chinese Glioma Genome Atlas (CGGA) of diffuse gliomas including IDH status and clinical information were downloaded from the CGGA website (http://www.cgga.org.cn) [9]. Patients' age of onset was not less than 18 years old; in addition, all the patients were primary lower grade gliomas in the cohorts. Overall survival (OS) was calculated from the date of diagnosis until death or the end of follow-up.

### 2.2. Weighted Correlation Network Analysis (WGCNA)

It is critical to construct a weighted coexpression network for identifying modules and for defining the intramodular connectivity. WGCNA is aimed at finding coexpressed gene modules and exploring the association between gene networks and phenotypes of interest. In coexpression networks, nodes correspond to genes, and connection strengths are determined by the pairwise correlations between expression profiles. In contrast to unweighted networks, weighted networks use soft thresholding of the Pearson correlation matrix for determining the connection strengths between two genes. Soft thresholding of the Pearson correlation preserves the continuous nature of the gene coexpression information and leads to results that are highly robust with respect to the weighted network construction method [10]. Firstly, the TCGA cohort of samples was clustered to remove the outliers ([Supplementary-material supplementary-material-1]). Finally, 508 cases of LGG were used for analysis. The power of 5 is interpreted as a soft threshold of the correlation matrix in the TCGA LGG RNA-seq cohort ([Supplementary-material supplementary-material-1]). Based on the resulting adjacency matrix, we calculated the topological overlap, which is a robust and biologically meaningful measure of network interconnectedness [11], that is, the strength of two genes' coexpression relationship with respect to all other genes in the network. Genes with highly similar coexpression relationships were grouped together by performing average linkage hierarchical clustering on the topological overlap. We used the Dynamic Hybrid Tree Cut algorithm to cut the hierarchal clustering tree and defined modules as branches from the tree cutting [12]. Then, we calculated the correlation between the module and the IDH status (*p* < 0.05 for the difference to be significant).

### 2.3. Statistical Analysis and Graphics

We crossed the genes associated with IDH obtained by WGCNA in the dataset. Differential expression analysis and correlation analysis were performed on the related genes enriched in the metabolic genes on the KEGG pathway in the intersection data of the dataset. Differential expression analysis was performed using a two-tailed *t*-test, and the measurement data were expressed as mean ± sd. Correlation analysis was performed using the Pearson method. In the IDH-WT gliomas, the identified differential metabolic genes were clustered by hierarchical cluster analysis. Univariate survival analyses were performed using the Kaplan–Meier estimator and the log-rank test. Multivariate Cox regression analyses were performed to identify independent prognostic factors. The random forest map was used to analyze the IDH classification.

Data were analyzed using R language, SPSS 21.0 software, and GraphPad Prism 7.0 software. WGCNA applied “WGCNA package.” Wayne diagram was drawn by “VennDiagram package.” *t*-test and survival analysis applied SPSS 21.0 software. Survival curves and histograms were drawn using GraphPad Prism 7.0 software. ^∗^*p* < 0.05, ^∗∗^*p* < 0.01, and ^∗∗∗^*p* < 0.001 were considered to be significant for all of the tests.

## 3. Results

### 3.1. Identification of Metabolic Genes Based on IDH Status

**Figure 1:**
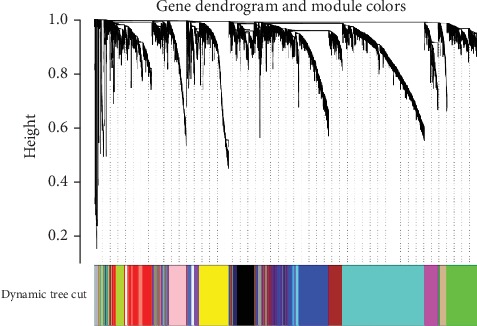
Cluster dendrogram based on dynamic tree cut. Networks were created from the weighted correlation matrices using WGCNA (Methods and Materials). Modules were assigned colors as indicated in the horizontal bar beneath the dendrogram. In the LGG dataset, 11 gene coexpression modules were detected, which were related to the IDH status. They were the black module, blue module, brown module, cyan module, green module, magenta module, midnight blue module, purple module, royal blue module, tan module, and yellow module.

From the TCGA LGG RNA-seq cohort, 11 gene modules with expression levels which were associated with IDH mutation were identified, respectively (Figure 1, [Supplementary-material supplementary-material-1]). A total of 5406 genes were identified in datasets. Based on the KEGG database: Kyoto Encyclopedia of Genes and Genomes (http://www.broadinstitute.org/gsea/c2.cp.kegg.v5.0.symbols), we screened out 309 metabolism-related genes, among which 220 genes had different expression in IDH wild type and mutant type (*p* < 0.001, [Supplementary-material supplementary-material-1]).

### 3.2. Screening Characteristic Attribute Genes Related to IDH Mutation Classification

**Figure 2:**
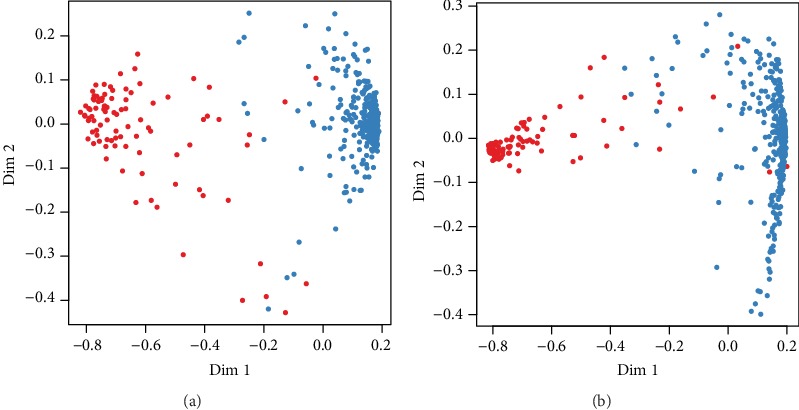
(a) 220 metabolic genes to detect the IDH wild-type group and IDH mutant group with the random forest prediction model. (b) The top 20 metabolic genes based on the value of MDG to detect the IDH wild-type group and IDH mutant group with the random forest prediction model.

In the TCGA LGG RNA-seq cohort, we constructed a random forest prediction model with the 220 metabolism-related differential genes. We selected the top 20 genes that contributed the most to the classification, according to the value of mean decrease in the Gini index (Table 1). Then, we used these 20 genes to construct a random forest prediction model again. 2/3 of the data was randomly selected as a training group and the rest as a test group. In the new model, the training group had a classification accuracy of 99.09%, a sensitivity of 98.90%, and a specificity of 100%. The OOB (out of bag) was 0.91%. And in the test group data, the classification accuracy was 97.78%, the sensitivity was 97.32%, and the specificity was 100%. The multidimensional scale plots of the IDH-MUT group and the IDH-WT group were clearly classified (Figure 2).

### 3.3. Differences in Fatty Acid Metabolism between IDH Mutants and Wild-Type Gliomas

**Figure 3:**
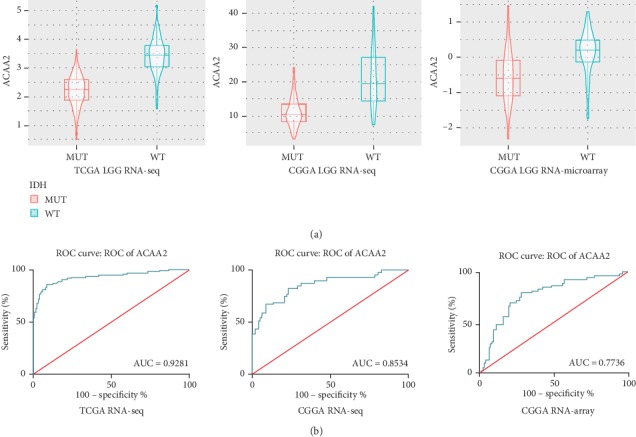
ACAA2 expression significantly different between the IDH wild-type (IDH-WT) and IDH mutant (IDH-MUT) groups. (a) The ACAA2 in IDH-WT and IDH-MUT showed significantly different expression RNA-seq pattern in TCGA, CGGA, and RNA microarray in TCGA (*p* < 0.05). (b) Corresponding ROC graph showed that the area under the ROC curve (AUC) in specificity of the RNA-seq pattern in TCGA, CGGA, and RNA microarray in TCGA was 0.9281, 0.8534, and 0.7736, respectively.

**Figure 4:**
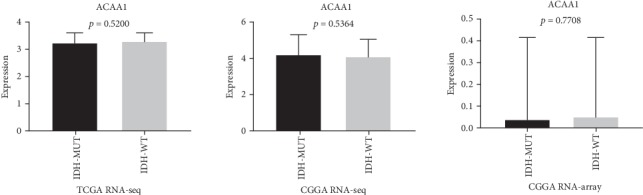
ACAA1 expression between the IDH-WT and IDH-MUT groups. The ACAA1 in IDH-WT and IDH-MUT showed no significantly different expression RNA-seq pattern in TCGA, CGGA, and RNA microarray in TCGA (*p* > 0.05).

The expression of fatty acid beta oxidation-related enzyme ACAA2 was significantly different between the IDH mutant and IDH wild-type gliomas. The expression of ACAA2 in IDH wild-type glioma was significantly higher than that in IDH mutant glioma, while participating in fatty acid beta. In order to verify the data in the CGGA chip and RNA-seq. A corresponding ROC graph is shown (Figure 3) in order to present the accuracy of classification using the differential expression of ACAA2 in the RNA-seq and microarray results in the CGGA and TCGA databases. There was no significant difference in the oxidized ACAA2 isoenzyme ACAA1 (Figure 4), suggesting that the IDH mutation is associated with the expression of ACAA2.

### 3.4. ACAA2 Can Be Used as a Prognostic Marker for IDH Mutant LGG

**Figure 5:**
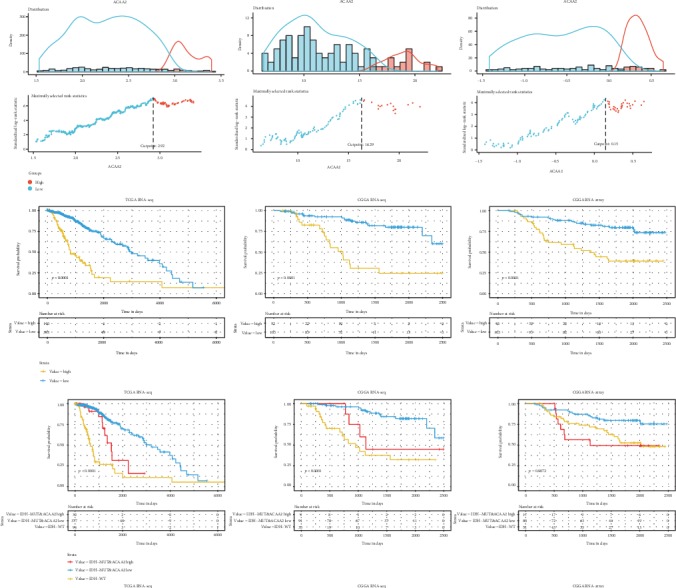
Clinical data showed that ACAA2 significantly affects the prognosis of LGG. “survminer R package” was used to find the cutoff value of ACAA2. The optimal cutoff value of ACAA2 was 2.92, 16.29, and 0.15, respectively, in the TCGA RNA-seq cohort, the CGGA RNA-seq cohort, and the CGGA RNA array cohort. The overall survival time (OS time) in the ACAA2 high expression group is significantly longer than that in the ACAA2 low expression group. And as for the IDH mutant group, ACAA2 lower expression also presented a better prognosis than those with ACAA2 high expression (*p* < 0.05).

ACAA2 in the TCGA and CGGA primary LGG dataset was grouped according to survival conditions and divided into ACAA2 high expression and low expression groups. Moreover, we also perform survival analysis by reclassification of LGG based on ACAA2 expression and IDH mutation. As the picture shows, even with IDH mutant LGG, the prognosis is still poor when ACAA2 is highly expressed (Figure 5). COX regression analyses of clinical risk factors (age of diagnosis, gender, grade, chemotherapy, and radiotherapy) and new classification (IDH-WT, IDH-MUT and ACAA2 high, and IDH-MUT and ACAA2 low) are shown in Table 2.

## 4. Discussion

Glioma is the most common form of malignant tumor in the human central neural system, which is believed to be closely associated with many factors, including biology, genomics, inflammatory, and metabolism factors [1, 13]. Inspired by the finding in many other cancers, metabolism factors had attracted much attention [9, 14]. In this present study, we use WGCNA method to analyze the transcriptome data of TCGA databases and found some interesting evidence to prove the importance of metabolism factors in glioma.

Based on IDH status, differentially expressed genes related to IDH mutants were identified. Differential expression analysis and correlation analysis were performed on the related genes enriched in the metabolic genes on the KEGG pathway in the intersection data of the dataset. We screened out 309 metabolism-related genes, among which 220 genes had different expression in IDH wild type and mutant type. Then, we selected the top 20 genes that contributed the most to the classification. Most of these genes are enzymes in the metabolism of tumor cells, such as HS3ST3B1, GLUD1, BCAT1, and ACAA2. The expression of fatty acid beta oxidation-related genes was significantly different. The expression of ACAA2 in IDH1 wild-type glioma was significantly higher than that in IDH1 mutant glioma, while participating in fatty acid beta. ACAA2 in the TCGA and CGGA primary LGG dataset was grouped according to survival conditions and divided into ACAA2 high expression and low expression groups. Performing survival analysis, we focus on reclassification of LGG based on ACAA2 expression and IDH1 mutation. Even with IDH1 mutant LGG, the prognosis is still poor when ACAA2 is highly expressed. On the basis of clinical risk factors, we created a new classification and divided lower grade gliomas into IDH1-WT, IDH1-MUT and ACAA2 high, and IDH1-MUT and ACAA2 low types.

The identification of mutations in the isocitrate dehydrogenase (IDH) genes, albeit in only a small percentage of gliomas a decade ago, has transformed our understanding of biology, genomics, and metabolism in gliomas. By far, three subtypes of IDHs were successively being discovered, namely, IDH1, IDH2, and IDH3 [1, 15, 16]. IDHs represent key enzymes within the tricarboxylic acid (TCA) cycle, and mutations of IDHs were believed to be involved in the metabolic processes of glucose, lipid, and amino acid in both physiology and pathology procedures [17]. Accumulating evidence indicates that mutations in canonical metabolic enzymes can promote the development of cancer [18]. Cancer-associated mutations in IDHs represent one of the most comprehensively studied mechanisms of IDH pathogenic effect. For gliomas, mutation at Arg^132^ of IDH1, and at the analogous codon Arg^172^ of IDH2, represents early initiating events that drive the evolution of low-grade glioma, including grade II to III astrocytoma and oligodendroglioma [16]. These mutations are also detected in grade IV glioblastoma (GBM), referred to as IDH1 mutant GBM, which account for ~10% of all grade IV clinical cases but are absent in pediatric high-grade malignancies and in nonglial subtypes of brain tumors [19]. Nevertheless, although we have learned that IDH mutations have a significant impact on metabolism processes of gliomas, we are still unclear about how IDH mutation regulates lipid metabolism processes in gliomas. And much still remains to be further elucidated to confirm the more detailed mechanism that how IDH mutations could affect lipid metabolism and the biological processes of gliomas.

Rapid growth and division are among the major characteristics of gliomas and other types of malignant tumors. Given the important role of lipids in cell membrane formation and signaling transduction, identification of the differences in lipid composition between gliomas and normal tissues, in order to find possible diagnostic and prognostic biomarkers for glioma patients, has been a long-term endeavor for biochemists. It was showed by Srivastava et al. [20] that cholesterol esters, formed by the esterification of cholesterol with long-chain fatty acids, have been shown to only be present in high-grade gliomas rather than lower grade gliomas as well as normal brain tissue, which indicated that lipid metabolism may differ markedly between different levels of gliomas. Recently, a growing number of research studies have focused on the lipid metabolism related to gliomas. Recently, the molecular mechanisms of regulation of lipid synthesis and reprogramming in glioma have been investigated. RTK/PI3K/Akt signaling has been shown to regulate lipid metabolism through upregulation of SREBP-1 transcriptional activity. Targeting fatty acid synthesis and reducing cellular cholesterol levels were shown to significantly inhibit GBM growth, particularly in EGFRvIII-expressing tumors [21]. Therefore, altered lipid metabolism is emerging as a potential therapeutic target in malignant gliomas.

**Figure 6:**
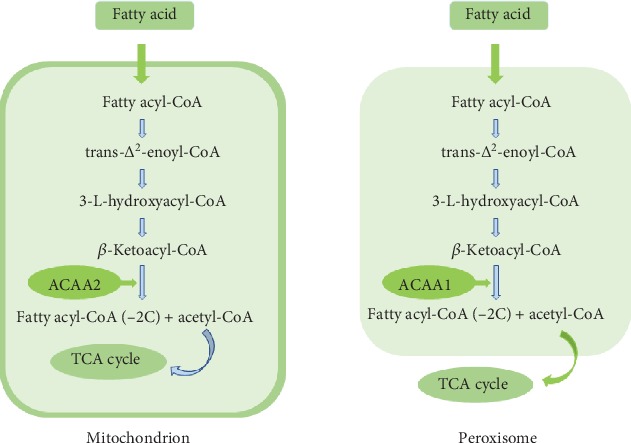
ACAA is one of the enzymes which catalyzes the last step of the beta oxidation pathway, an aerobic process breaking down fatty acids into acetyl-CoA. ACAA2 is located in the mitochondrion, while ACAA1 in the peroxisome.

ACAA2 is an important enzyme that is involved in the beta oxidation of lipid acids. It is capable of catalyzing the beta oxidation process with its isoenzyme, namely, ACAA1 in a complex mechanism [6]. Researchers have found that ACAA2 was associated with lipid concentrations and cardiovascular risk factors as well as the function of blood low-density lipoprotein cholesterol, high-density lipoprotein cholesterol, or triglycerides in humans. However, until now, no related research studies provided any evidence that ACAA2 could have a crucial role during cancer processes. As presented in our study, we found that ACAA2 was among the differential expressed genes of LGG, which indicate that ACAA2 may be important in the development and outcome of LGG. Moreover, what makes us curious is that the expression of ACAA2 is significantly different between the IDH mutant and wild-type groups, while the expression of ACAA1 is not, which indicated that ACAA2 might play a crucial role in the lipid acid oxidation process in glioma and further affect the occurrence and development of glioma. It is suggested that targeted therapy for ACAA1 in IDH mutation gliomas may be a new promising and effective target for gliomas (Figure 6).

However, even though we found some evidence which proved a clear relationship between ACAA2 and metabolism in the biological process of glioma, experiments in a molecular level were still needed. Thus, in order to further verify our findings, in our future study, more cell biology and biochemistry experiments would be conducted so that we would find more valid data in order to finally reach a conclusive discovery.

## 5. Conclusion

In conclusion, in our study, we revealed that many molecules expressed differently between IDH-MUT and IDH-WT in lower grade gliomas by bioinformatics methods. And among these genes, ACAA2, a crucial factor which regulates lipid acid beta oxidation, might have a vital role in the complex mechanism of metabolism processes in gliomas and might also be a new potential target for gliomas' comprehensive treatment.

## Figures and Tables

**Table 1:** The top 20 metabolic genes based on the value of MDG.

Gene name	Gene full name	Mean decrease Gini
B3GNT7	Beta-1,3-N-acetylglucosaminyltransferase 7	10.34689677
ACSS3	Acyl-CoA synthetase short-chain family member 3	7.909816
DCTD	dCMP deaminase	7.36251499
PPCS	Phosphopantothenoylcysteine synthetase	6.42529875
PLA2G5	Phospholipase A2 group V	6.36536675
PIPOX	Pipecolic acid and sarcosine oxidase	6.12854856
ACAA2	Acetyl-CoA acyltransferase 2	5.55485439
GALNT13	Polypeptide N-acetylgalactosaminyltransferase 13	5.54486482
OPLAH	5-Oxoprolinase, ATP hydrolysis	5.04530335
HS3ST3B1	Heparan sulfate-glucosamine 3-sulfotransferase 3B1	4.89715727
CHST6	Carbohydrate sulfotransferase 6	4.58671345
PNPLA4	Patatin-like phospholipase domain containing 4	4.40125265
FUCA2	Alpha-L-fucosidase 2	3.22170785
BCAT1	Branched chain amino acid transaminase 1	3.00358464
GLUD1	Glutamate dehydrogenase 1	2.85948492
CBR1	Carbonyl reductase 1	2.84089168
GALM	Galactose mutarotase	2.44693901
B3GNT5	Beta-1,3-N-acetylglucosaminyltransferase 5	2.15619286
ENTPD2	Ectonucleoside triphosphate diphosphohydrolase 2	2.15531693
CA3	Carbonic anhydrase 3	2.02087863

**Table 2:** Clinical risk factors and new classification (IDH-WT, IDH-MUT and ACAA2 high, and IDH-MUT and ACAA2 low) COX regression.

Dataset	Variable	*p*- value	HR	HR 95% CI	
TCGA seq	New group	<0.001	2.912	1.986	4.272
	Age > 40	<0.001	0.412	0.274	0.620
	≤40				
	Grade	<0.001	2.620	1.730	3.967

CGGA seq	New group	<0.001	2.170	1.460	3.225
	Grade	<0.001	5.913	2.744	12.742

CGGA array	Grade	<0.001	5.507	3.086	9.828
	Age > 40	0.044	0.550	0.308	0.983
	≤40				

COX regression: step forward (likelihood ratio). Missing the information on surgical resection.

## Data Availability

The data used to support the findings of this study are included within the article.

## Supplementary Materials

Supplementary 1 Figure S1: sample clustering to detect outliers in the TCGA LGG RNA-seq cohort. Figure S2: scale independence and mean connectivity of the TCGA LGG RNA-seq cohort. Figure S3: correlation between different module genes and IDH status.

Supplementary 2 Table S1: 220 metabolic genes with significant differences in the IDH wild-type and mutant groups.
