# Supplementary material for: Transcriptomic Analysis of Glioma Based on IDH Status Identifies ACAA2 as a Prognostic Factor in Lower Grade Glioma
Source: Biomed Res Int. 2020 Mar 21;2020:1086792. doi: 10.1155/2020/1086792 (PMC7115055; doi:10.1155/2020/1086792)
Supplement: Supplementary 1 — Figure S1: sample clustering to detect outliers in the TCGA LGG RNA-seq cohort. Figure S2: scale independence and mean connectivity of the TCGA LGG RNA-seq cohort. Figure S3: correlation between different module genes and IDH status. [file 1086792.f1.docx]

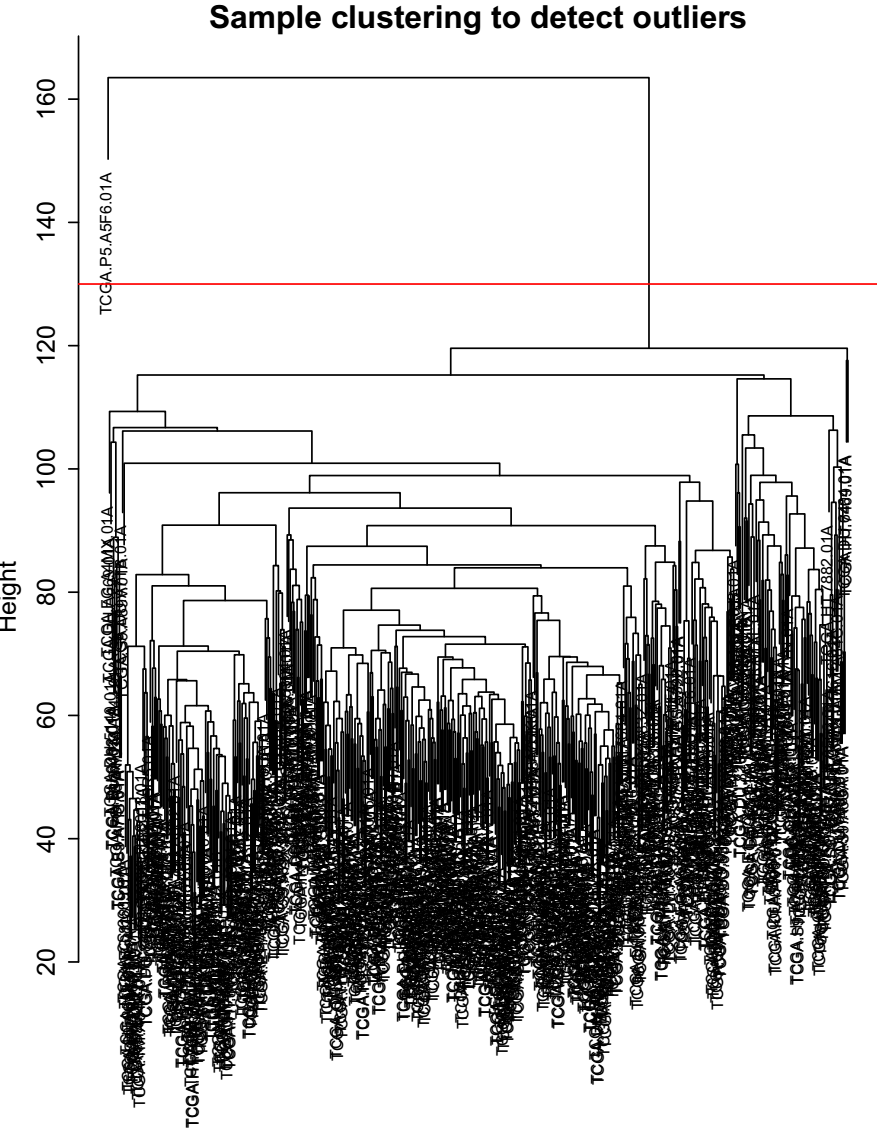


**Figure S1: Sample clustering to detect outliers in the TCGA LGG RNA-seq cohort**

The TCGA LGG RNA-seq cohort result showed that samples in TCGA LGG RNA-seq can be clustered into different subgroups.


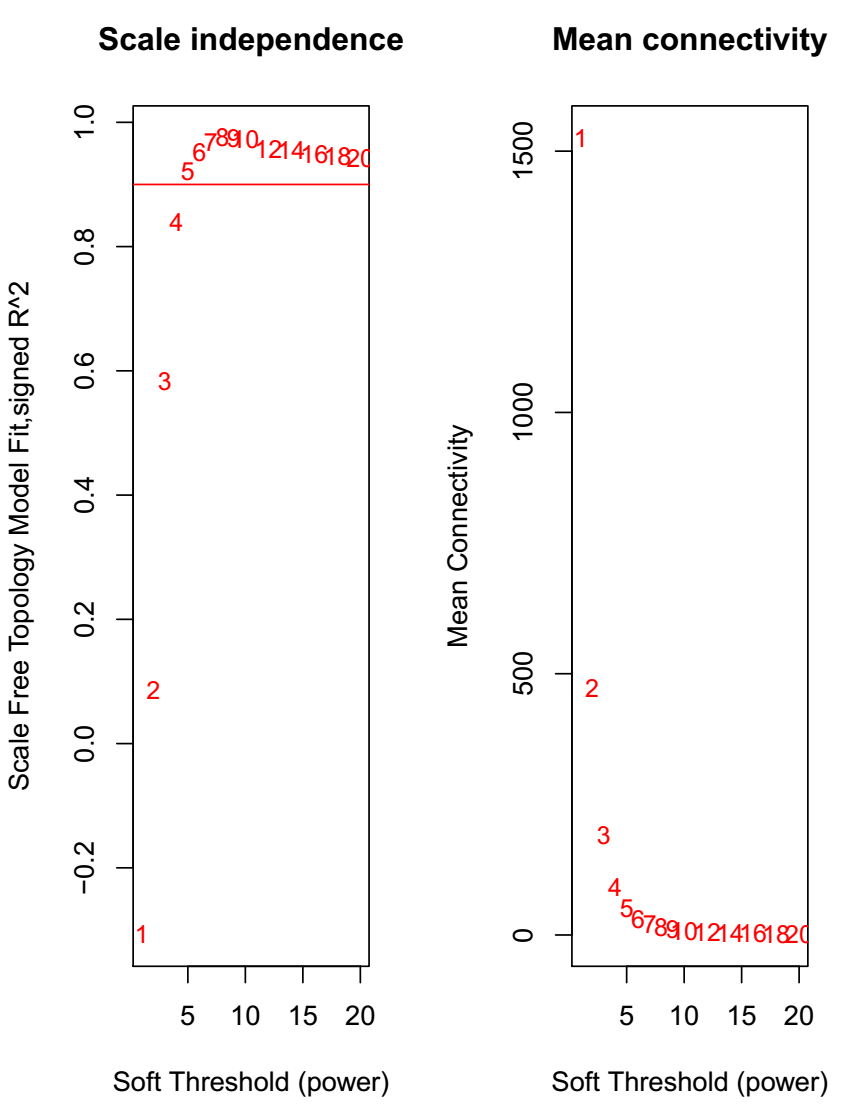


**FigureS2: Scale independence and Mean connectivity of TCGA LGG RNA-seq cohort**

The relationship between Scale independence and soft threshold, as well as mean connectivity and soft threshold showed the cluster pattern of TCGA LGG RNA-seq cohort samples. Correlation coefficient should be at least 0.9. The power of 5 was interpreted as a soft-threshold of the correlation matrix.


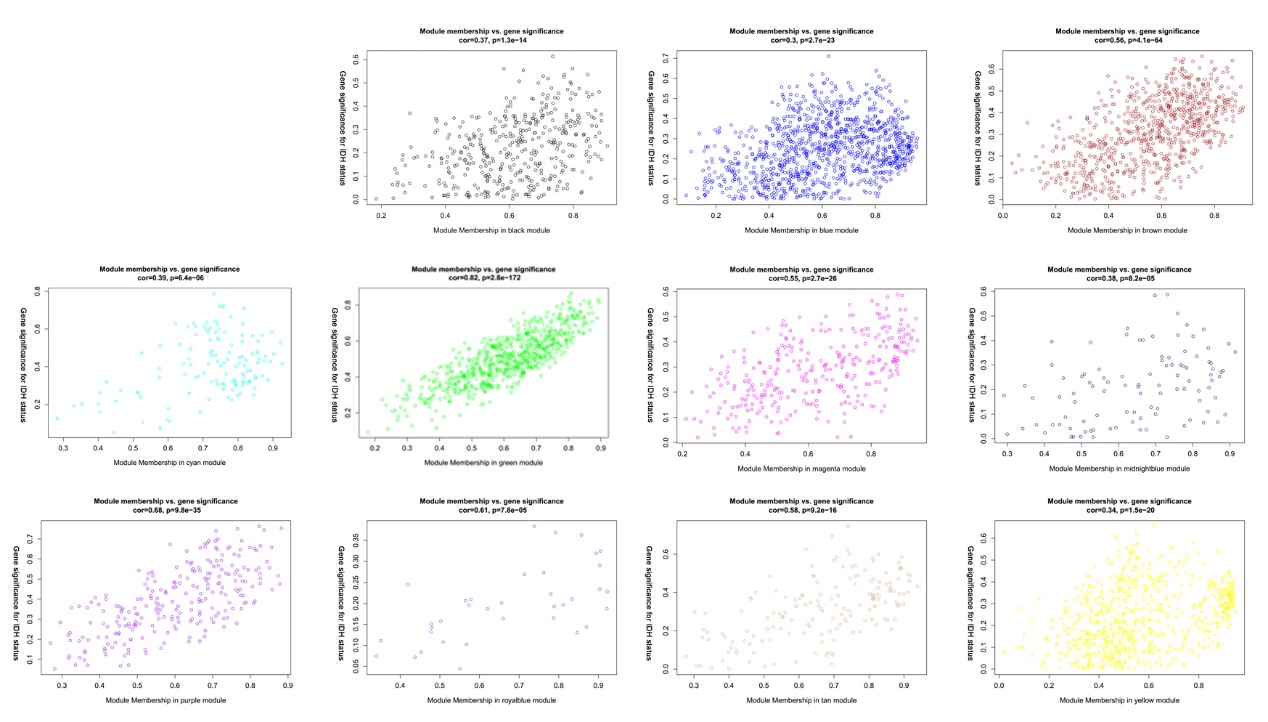


**Figure S3: Correlation between different module genes and IDH status.**
